# Fiberglass Grids as Sustainable Reinforcement of Historic Masonry

**DOI:** 10.3390/ma9070603

**Published:** 2016-07-21

**Authors:** Luca Righetti, Vikki Edmondson, Marco Corradi, Antonio Borri

**Affiliations:** 1 Department of Mechanical and Construction Engineering, Northumbria University, 209 Wynne-Jones Building, Newcastle upon Tyne NE1 8ST, UK; luca.righetti@northumbria.ac.uk (L.R.); edmondson@northumbria.ac.uk (V.E.); 2 Department of Engineering, University of Perugia, 92 Via Duranti, Perugia 06125, Italy; antonio.borri@unipg.it

**Keywords:** Textile Reinforced Mortars (TRM), GFRP grids, ageing tests, mechanical tests, masonry walls, masonry tile vaults, ring beam reinforcement

## Abstract

Fiber-reinforced composite (FRP) materials have gained an increasing success, mostly for strengthening, retrofitting and repair of existing historic masonry structures and may cause a significant enhancement of the mechanical properties of the reinforced members. This article summarizes the results of previous experimental activities aimed at investigating the effectiveness of GFRP (Glass Fiber Reinforced Polymers) grids embedded into an inorganic mortar to reinforce historic masonry. The paper also presents innovative results on the relationship between the durability and the governing material properties of GFRP grids. Measurements of the tensile strength were made using specimens cut off from GFRP grids before and after ageing in aqueous solution. The tensile strength of a commercially available GFRP grid has been tested after up 450 days of storage in deionized water and NaCl solution. A degradation in tensile strength and Young’s modulus up to 30.2% and 13.2% was recorded, respectively. This degradation indicated that extended storage in a wet environment may cause a decrease in the mechanical properties.

## 1. Introduction

The protection of the richness and diversity of the architectural heritage of Europe constitutes a priority to the Member States [1]. The conservation and preservation of historic buildings, necessitates repair and retrofitting and is particularly challenging in earthquake prone-areas. Accordingly, technology advancements which widen the choices of restoration methods are important [2,3,4].

Earthquake damage in many parts of the world has demonstrated that historic masonry buildings have been designed with no regard for the effects of horizontal loading produced during a seismic event. As the tensile strength of masonry may be assumed to be equal to zero, researchers have studied the use of composite materials in order to reinforce masonry and to resist seismic actions.

It is known that fiber-reinforced composites (FRPs) are composite materials made of artificial or natural fibers embedded in a polymeric matrix. These exhibit several positive characteristics which make them suitable as structural reinforcing elements. FRPs are characterized by high tensile strength in the fibers’ direction and by linearly-elastic response up to failure. The polymeric matrix has two principal functions: transfers the load to the fibers and protects them from degradation due to environmental effects. Polymeric organic matrices are usually made of epoxy resins. Both organic matrices and some composite fibers (i.e., carbon and Kevlar) are produced from fossil fuels. 

Fiberglass (GFRP) is a type of fiber-reinforced polymer where the reinforcement fiber is specifically glass fiber. This has been used in the construction industry since the beginning of the 1980s [5,6]. Chiefly, its application was widely researched as a strengthening and rehabilitating technique to improve the performance of reinforced concrete structures, with less research undertaken for masonry construction. However, FRP cost (both the composite material and the epoxy adhesive) and the limited improvement compared with conventional retrofitting methods, curtailed a widespread adoption for reinforcement of non-historic masonry constructions. Investigation of composites application to masonry structures first occurred in USA in the 1980s and involved the use of GFRPs epoxy bonded to the masonry substrate.

During 1995 to 2015 a series of experiments was carried out at several research centers across Europe and US to assess a range of different strengthening techniques for historic masonry using GFRP materials [7,8,9,10]. Initially all these methods involved the use of epoxy adhesives to bond the composites to the masonry substrate. The principal focus of attention was the implementation of near surface mounted wall reinforcement using GFRP sheets and plates, usually having a unidirectional fiber composition, providing resistance against in-plane seismic actions. Vaults and arches also received considerable attention, with many papers dealing with GFRP reinforcements applied at intrados or extrados. Other papers have considered methods for reinforcing stone or brick-masonry columns by producing a confinement effect using wrappings of glass fibers.

A more innovative approach has been recently proposed in several studies. In the early 2010s, the use of non-organic matrices/adhesives was considered with the aim to foster improved long-term behavior, provide reinforcement reversibility, reducing the energy consumption on production of construction materials, meet the requirements of conservation bodies and use more compatible reinforcement materials with historic masonry [11,12,13]. The use of lime- or low cement-based renders also allow the walls to breathe naturally and facilitates future eventual removal of the composite reinforcement. The adoption of non-organic matrices raises inherently less concerns regarding durability, product cost and health and safety restrictions compared to organic adhesives. This type of reinforcement can be also regarded as sustainable as it falls within the sustainable restoration techniques for historic constructions [14,15]. In 1987, The World Commission on Environment and Development in, Our Common Future, penned the now classic definition of sustainable development as “development which meets the needs of the present without compromising the ability of future generations to meet their own needs”. This definition has been recently applied to restoration and conservation of historic masonry when non-invasive methods, reversible interventions and sustainable materials are used [16,17].

For example, in an attempt to avoid problems associated with the use of epoxy resins, an innovative composite material, known as Textile Reinforced Mortar (TRM) was developed. TRM has been used over the last decade for seismic retrofitting of structures, particularly concrete and masonry structures [18,19,20]. While studies have shown that TRM are more effective in increasing the deformation capacity of structures, they are generally less effective in reinforcing historic masonry structures compared to FRP, due to a weaker bond of inorganic mortars with masonry substrates compared to epoxy resins. Non-organic matrices are not well established in applications involving the bonding of composite materials on masonry substrates. Their mechanical properties are also significantly weaker compared to epoxy adhesives.

In this paper, the fields of applications of TRM reinforcements of existing masonry elements are discussed and the results of previous experimental works are concisely reported and analyzed. A review of the recent developments in the field of retrofitting of historic masonry structures with TRMs is reported in the first part of this article. This paper also addresses the problem of the durability of GFRP grids exposed to various environmental conditions. Mechanical degradation due to ageing effects is an important aspect for composite materials, especially for glass-based composites [21,22,23,24]. Several specimens subjected to artificial ageing treatments have been tested in order to study the degradation mechanism in terms of tensile strength and stiffness. This study is aimed at giving a contribution to the existing literature on this specific topic. The resistance of GFRPs is usually affected by their exposure to environmental changes or degrading agents, such as alkaline agents and moisture variation [25,26,27]. The deterioration of matrices is due to hydrolysis, swelling and plasticization in water environments. Furthermore, this makes the interface between the fibers and the matrix weak, and consequentially produces a decrement of the properties of components of the masonry structure. Although the effect of these changes has been well studied in the past, further studies are required on the long term behavior of GFRP materials, especially when under new forms like grids or nets, not previously studied in the past. 

Several studies on the use of GFRP materials on historic masonry structural elements are limited to defining the improvements in terms of capacity and stiffness of reinforced masonry elements without due consideration of the long term behavior and the durability of the masonry. For example, GFRP grids, inserted into an organic matrix (lime- or cement-based mortar), can coexist with extremely high pH values, due to the hydration process of lime, that could potentially produce damaging of the glass fibers. 

Gangarao and Vijay [28] exposed glass and polyester composite materials samples to different treatments in water solutions. Uomoto [29] considered the susceptibility to dissolution in alkaline environment of the glass fibers. Lab studies on FRP materials exposed to different treatments in water solutions exhibited the possibility of sudden reduction of their mechanical properties. It is therefore fundamental to examine the effect and consequence of environmental factors on long-term performance of the FRP material.

## 2. GFRP Grid

**Figure 1:**
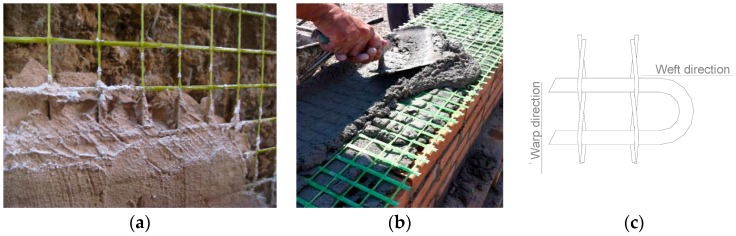
(**a**) 66 × 66 mm^2^ GFRP grid; (**b**) 33 × 33 mm^2^ GFRP grid; (**c**) schematic layout.

The TRM material used in the investigation is made up of an inorganic lime- or low cement-based mortar and GFRP grid. While the mortars are different both in composition and mechanical properties, the same GFRP grid has been used to reinforce all masonry structural elements described in Section 3. The GFRP grid is a commercially available product (Figure 1). It is characterized by a mesh size of 33 × 33 mm^2^ or 66 × 66 mm^2^ and continuous fiber filaments embedded in thermosetting epoxy vinyl ester resin matrix. The meshes are manufactured using fiberglass, with AR-glass (Alkali Resistant) reinforcement and have a zirconium content equal to or greater than 16%. The weight density of the 66 × 66 mm^2^ and 33 × 33 mm^2^ grids is 0.5 and 1 kg/m^2^, respectively.

## 3. Possible Applications for TRM Reinforcement

Previous work undertaken by the authors in recent years has considered the application of fiberglass grid-reinforcement to three structural elements—masonry wall panels, tiled vaults and finally masonry ring beams a typical solution deployed to retrofit masonry buildings. This section reviews the results of previous investigations undertaken utilizing GFRP grids as a means of enhancing the performance of the three retrofitted structural elements. The last section of this paper is devoted to considering the mechanical behavior of the GFRP mesh after ageing, an area not widely considered to date by researchers. The innovative results of an extensive experimental campaign on the long-term behavior of the GFRP mesh under artificial ageing will be presented and discussed in detail.

### 3.1. In-Plane Reinforcement of Wall Panels

A shear reinforcement is often needed to increase the lateral capacity of masonry wall panels. A series of 17 on-site shear tests were completed on wall panels from historic buildings at three locations in Italy: Colle Umberto, San Felice and Pica Alfieri. The results of these tests are described in detail in [30], and the main results are summarized in Table 1. Wall panels have been reinforced using the 66 × 66 mm^2^ GFRP grid described in Section 2, while a ready-to-use low cement-based mortar was applied to bond the grid to the masonry surface. The average 30-day compressive strength of mortar was 21.36 MPa. A four-part code comprised of both characters and indices has been used to uniquely identify each test. The first letter indicates the test type (CD = diagonal compression, SC = shear-compression), the second is a progressive number identifying individual panels, the third notes the location where the test was undertaken (U = Colle Umberto, S = San Felice, P = Pica Alfieri) and the four character identifies the type of shear strengthening undertaken (OR = unreinforced panel, IP = preventive reinforcement, IR = panel repaired).

Virgin masonry wall panels were reinforced or repaired using TRM materials in order to study the effectiveness of the retrofitting method as a preventive reinforcement or a repair. In San Felice building, a shear test was performed on an unreinforced masonry panel, and shear strength of 0.062 MPa was measured (SC-18-S-OR). During testing cracks were recorded in the unreinforced panel, only in the mortar joints along the compressed diagonals. The introduction of TRM reinforcement altered the failure behavior of the panel. Shear cracks still formed in the retrofitted masonry panel along the compressed diagonals of the lower and upper square semi-panels, but occurred following the development of horizontal cracks due to in-plane bending action at the midpoint of the panel. The shear tests completed in San Felice demonstrated an increase in shear strength from 0.020 to 0.086 MPa or the unreinforced (CD-09-S-0R) and preventative reinforced (CD-11-S-1P) respectively. 

A greater number of tests were performed on the masonry of the Colle Umberto building (Table 1). Comparable readings of shear strength where obtained for the unreinforced rough-hewn stone panels from the diagonal compressive tests, with results ranging from 0.018 to 0.021 MPa. The maximum values were recorded for the test specimens having a thickness of 600 mm, taken from the oldest masonry of the building, dating from the 19th Century. The shear strength results obtained from the unreinforced panels in Colle Umberto, had a similar magnitude to the shear strength readings from the solid bricks at the San Felice building of 0.020 MPa. Additionally, the cracks produced in the unreinforced panels at Colle Umberto had failed again exclusively in the mortar joint, through the entire thickness of the wall panel along the compressed diagonal. 

**Figure 2:**
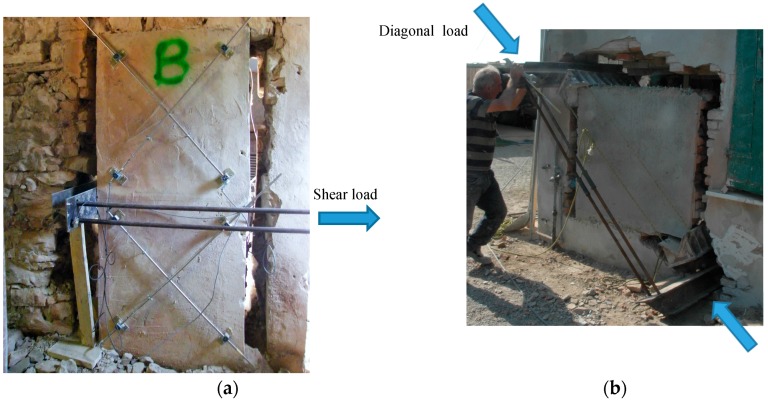
(**a**) Shear compression test; (**b**) diagonal compression test.

At Colle Umberto building shear tests completed after reinforcement had been applied to both retrofit virgin masonry panels for conservation purposes (preventative reinforcement), and to repair pre-damaged masonry, demonstrated substantial improvement in shear performance. A shear strength of 0.162 MPa (CD-07-U-IP) was measured for the panel with preventive reinforcement and 0.209 MPa (CD-08-U-IR) for the repaired panel, compared to a shear strength of 0.018 MPa in the same unreinforced panel: An increase varying between 800% and 1060% (Figure 2). 

The results of the shear tests for the reinforced Colle Umberto 20th-century masonry panel demonstrated similar improvements in shear strength. The test on the unreinforced panel measured a shear strength of 0.032 MPa (TC-16-U-OR) compared to a reinforced strength of 0.236 MPa (TC-20-U-1P): An increase of 638%. Improvements in the shear strength of the repaired panel were also measured; the tested panel recorded a shear strength of 0.173 MPa, an increase of 440%.

### 3.2. Reinforcement of Tiled Vaults

Many historic constructions are characterized by vaulting systems. These structural elements often need to be retrofitted to resist horizontal and vertical loads and the application at extrados of composite reinforcement is an interesting solution. In this investigation, 17 test specimens were constructed with a segmental arch shape, using methods typical of those employed for many historic bridges, involving the use of timber centering formwork as a temporary support. The experimental details are given in [31]. Geometrically, the specimen arches had a span of 2000 mm with a corresponding height of 700 mm above springer level. The unreinforced tile arches were built using flat rectangular tiles in two or three layers, having an overall thickness of 80 or 130 mm, respectively. 

**Figure 3:**
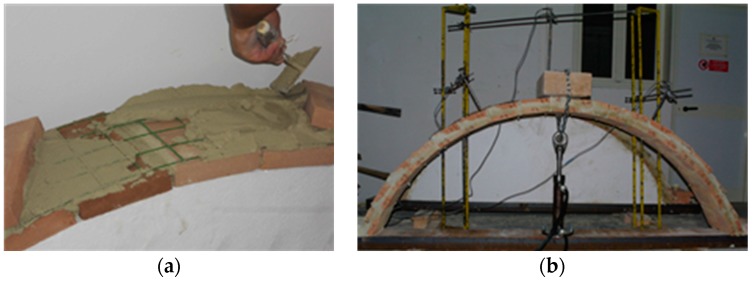
(**a**) Reinforcement application; (**b**) test arrangement.

Twelve specimens were reinforced within the mortar bed joints with TRM using the 66 × 66 mm^2^ GFRP grid described in Section 2. Once the bottom tile layer was in place a first layer of reinforcement was directly applied. The GFRP grid was cut to length of 3000 mm and width of 300 mm, covering the top side of the arch length and width entirely. Afterwards, further layers of TRM were applied to mortar joints as required dependent on the arch’s construction (double-layer arch or triple-layer arch) following an identical procedure (Figure 3a). Additionally, for several specimens, reinforcement was also applied to the outer surface of the arch. Two types of mortar have been used: Mortar type 1 has a flexural and compressive strength of 0.58 and 0.16 MPa, respectively. For mortar type 2, these are 5.25 and 1.96 MPa.

Specimens were tested by applying a vertical compression load at vault crown (Figure 3b). Test results are reported in Table 2. All test specimens are identified by a three index code, in which the first character indicates the number of arch rings (DR, double-layer arch; TR, triple-layer arch); the second character designates the type of strengthening completed (UT, unreinforced; IT, reinforced vault with GFRP grid placed into the mortar bed joints; OT, reinforced with GFRP grid placed both into the mortar bed joints and on the outer surface of the arch); and the third index indicates the identification number of the specimen.

Test results have also demonstrated that the use of composite TRM strengthening increases the load capacity of arch specimens. The capacity of the arches strengthened with TRM introduced solely into the mortar bed joints was increased by 448% from 0.15 to 0.82 kN for arch DR.IT.01, built with a weak mortar, and by 1.57 to 3.52 kN for arch DR.IT.02 built with a single-component high-strength repair mortar (Type 2). The use of GFRP grid inserted both into the mortar bed joints and on the outer surface of the arch (OT arches) permitted the attainment of capacity increases ranging between 376% and 569%. A comparison between arches with equivalent mortar type (Type 2) highlighted that OT arches provided an ultimate load capacity approximately 148% and 48% larger than those recorded for DR.IT and TR.IT specimens, respectively. The failure mode of reinforced specimens was due to the relative separation of the layers of tiles and GFRP reinforcement, while, for unreinforced specimens, this was mainly due to the formation of a 4-hinge mechanism.

### 3.3. Ring Beam Reinforcement

The adoption of a ring beam at the head of masonry or brick walls to promote construction box-like behavior and prevent wall out-of-plane collapse mechanisms is an accepted and effective practice against horizontal actions. TRMs can be also applied here, facilitating at roof level the introduction of a composites-reinforced masonry ring beam to provide a connection between adjacent walls and between walls and floors. The method consists in the application of a masonry ring beam reinforced at the mortar bed-joints with the GFRP grid using recycled or new stone or bricks. This also allows to keep the fair-faced aspect of the masonry as the reinforcement is completely embedded into the mortar joints. As part of a larger previous study, described in detail in [32], six full-scale masonry beams were constructed and subject to bending tests. Four stonemasonry specimens (length 5 m), were constructed from 3 layers of stones and 4 layers of GFRP grid embedded in a ready-to-use mix hydraulic lime-mortar, to achieve an overall cross-section of 0.5 × 0.5 mm^2^. Similarly, two brickmasonry specimens (length 5 m), were constructed from 4 layers of clay bricks and 5 layers of GFRP mesh embedded in a ready-to-use cement-based mortar to achieve an overall cross-section of 0.4 × 0.33 mm^2^. The 55 × 120 × 250 mm^3^ clay bricks were hollow with a 36% void area. 

**Figure 4:**
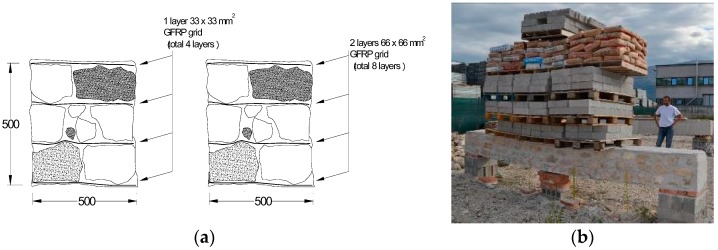
Ring beams under loading: (**a**) grid arrangement for stonemasonry beams; (**b**) P5-G33-V.

Two different sizes of GFRP grid were used in the tests undertaken, having a rigid square grid size of 33 × 33 mm^2^ or 66 × 66 mm^2^, as described in Section 2. For ring beam test samples P5 and P6 a 33 × 33 mm^2^ GFRP mesh was adopted, whilst a 66 × 66 mm^2^ mesh was installed in ring beam test specimen P7 and P8. In order to have the same quantity of composite material, when the 66 × 66 mm^2^ grid was used, 2 overlapping grids have been inserted into the horizontal mortar joints (Figure 4a). The strength of the lime-based mortar (type CM) and the cement-based mortar (MI) used for the stone- and brick-masonry beams respectively, was determined by compression tests on cylindrical samples. The mean value of the compressive strengths was 5.99 and 10.61 MPa, for mortar CM and MI, respectively. 

It is well understood that historic masonry has high compressive strength, but also has very low tensile strength unless reinforced. Accordingly, for a span over 4 m it was structurally not possible to construct unreinforced specimens of similar geometry as those reinforced as a control to compare results. Considering purely the self-weight of such a control beam, the stress on the unreinforced beam’s tension side would be much higher than the masonry tensile strength. A masonry beam with the dimensions used in this investigation thereby cannot stand unreinforced over a span of 4 m, especially when it is made using a lime-based mortar. 

The reinforced masonry ring beams were tested by applying a bending load perpendicularly and parallel to the mortar bed joints and GFRP grid. The ends of each beam were simply supported on two 0.5 × 0.5 × 0.25 mm^3^ masonry blocks. The bending load was applied to the beams using concrete cubes and/or cement bags. The deflections and possibility of the beam twisting has been taken into account by applying linear variable displacement transducers on both sides of the beams (Figure 4b).

The test results for the beams specimens are presented in Table 3. It can be noted that the application of the composite-reinforcement is able to provide masonry with the needed tensile strength to resist severe bending loads. Each bending test is identified with a code comprised of three indices, the first indicates the masonry type (P and L, for stone- and brick-masonry, respectively) and the beam’s identification number, the second the type of strengthening (G33 = GFRP mesh with a grid size of 33 × 33 mm^2^; G66 = GFRP mesh with a grid size of 66 × 66 mm) and the last one the direction of the bending loads with regard to the mortar bed-joints (V or H for bending loads parallel or perpendicular to the mortar bed joints, respectively). In Table 3, maximum mid-span bending moments produced by both self-weight (*M*_w_) and by external loads (*M*_Load_) are listed. The bending moment produced by the self-weight has been obtained from the masonry weight density; 2140 and 1464 kg/m^3^ for stone- and brick-masonry beams, respectively.

The overall bending capacity of the stone-masonry and brick-masonry ring-beams was demonstrated to be significantly high enough to enable viable practical performance within a retrofitted building. The maximum mid-span bending moment achieved was 54.73 kNm arising from a test failure load of 62.9 kN applied to ring beam P7-G66-V. Failure initiated at mid-span with the opening up of vertical cracks, which amplified with progressive load and horizontal cracks at the bed joints opened near the beam’s ends. For the brick-masonry beams, specimen L9-G33-V achieved a bending moment of 35.87 kNm, whilst the test for L10-G33-H was stopped with an external load of 38.3 kN without the beam having reached failure. 

## 4. Ageing Tests

It is known that the glass fiber, which is used in GFRP reinforcement, is susceptible to attack by OH-ions. Therefore, an important task for the resin is to act as a barrier, defending the glass fibers from damaging agents. However water and possibly alkalis can penetrate over time the resin and eventually attack the fibers, the fiber/resin interface or the resin itself through plasticization, hydrolysis and other mechanism of degradation, which may cause irreversible changes in the resin structure.

**Figure 5:**
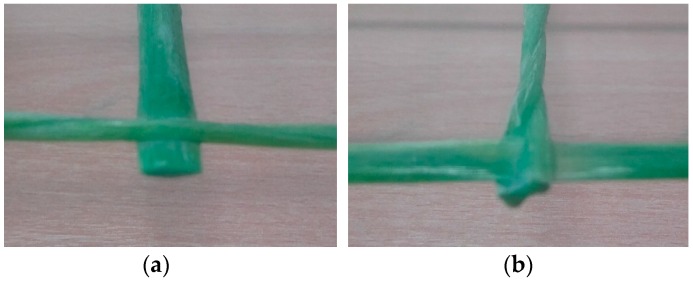
66 × 66 mm^2^ GFRP grid: (**a**) weft direction (SR type); (**b**) warp direction (SC type).

To determinate the effect of accelerated alkaline corrosion (ageing) on the strength characteristics of the material, GFRP specimens have been subjected to different ageing treatments to evaluate any change in their mechanical characteristics. Specimens were cut from the warp and weft directions of the 66 × 66 mm^2^ GFRP mesh, using a diamond saw. The glass fibers in the two directions present different cross section shapes: Rectangular-shaped in the weft direction, and made of two twisted circular-cross section cords in the warp direction (Figure 5).

**Figure 6:**
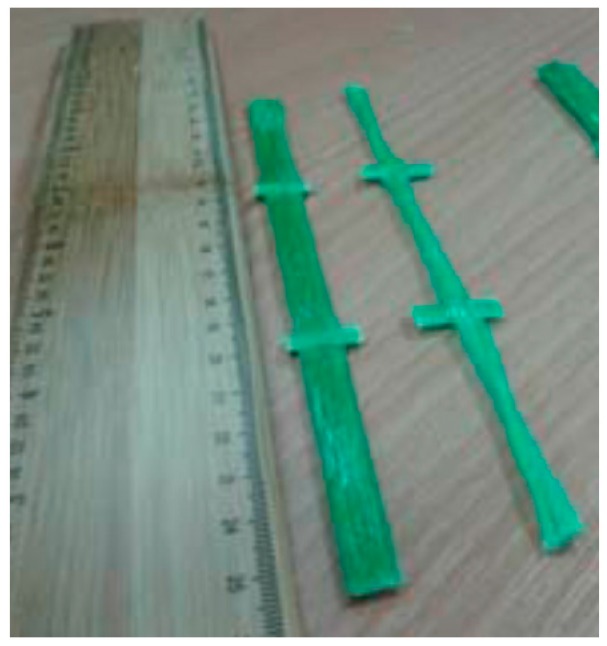
Specimens obtained from the 66 × 66 mm^2^ GFRP grid.

**Figure 7:**
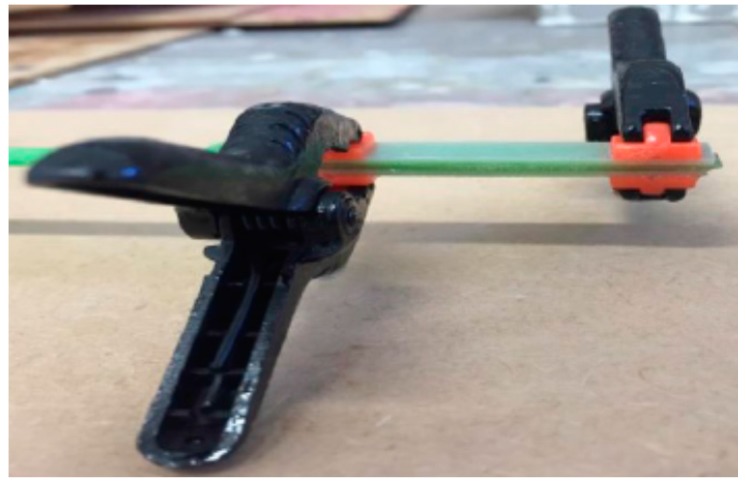
Detail of the epoxy tabs glued at both ends of the specimens.

SC and SR index have been adopted to identify circular (warp) and rectangular (weft) cross-sections characterized by a dry glass fiber cross sectional area of 3.8 mm^2^ in both directions and a weight density of 0.5 kg/m^2^. Specimens have been tested in tension (Figure 6), in accordance with ASTM D3039 standard [33], using an Instron Tensile Machine model 3382. The machine was equipped with a 100 kN load cell and a contact extensometer (using a 50 mm gauge). To minimize stress concentration near the grip zone fiberglass sheet packing pieces (tabs) were glued with epoxy resin at both ends of the specimen (Figure 7). All tensile tests were conducted with crosshead speed of 0.2–0.3 mm/min at room temperature of 21 ± 2 °C and humidity equal to 50%.

### 4.1. Untreated Specimens

**Figure 8:**
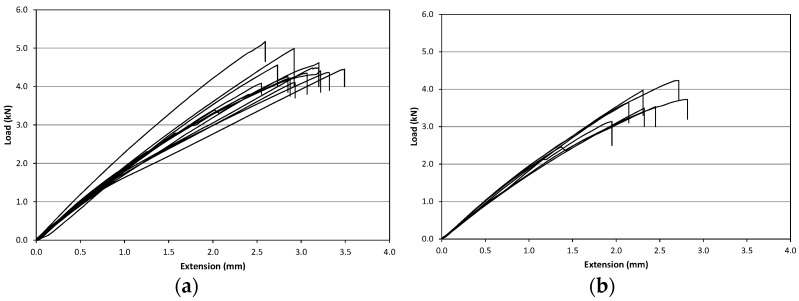
Load-extension curves for untreated specimens: (**a**) SR-series; (**b**) SC-series.

From the test results, the tensile strength and the Young’s modulus, the stress-strain responses are calculated. Both tensile strength and Young modulus were derived using the dry glass fiber cross sectional area value (3.8 mm^2^). 22 un-treated GFRP specimens were tested. Standard Deviation (SD) and mean values were determined as given in Table 4. These results have been reported exclusively for the purpose of qualitative evaluation of the decrease in the mechanical property due to the different treatments described in the following paragraphs. During the tests, GFRP specimens demonstrated an approximately linear behavior up to failure (Figure 8a,b). The Young’s modulus, E, was calculated from the stress-strain data by using an extensometer in a load range of 0.2–2.0 kN.

**Figure 9:**
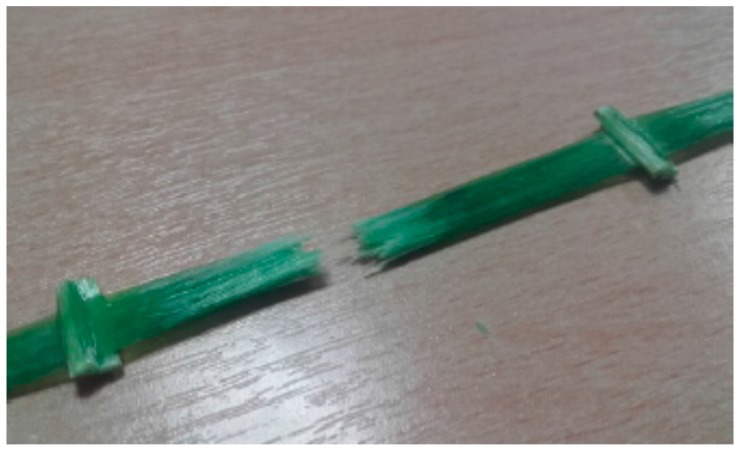
Catastrophic collapse of the GFRP sample.

**Figure 10:**
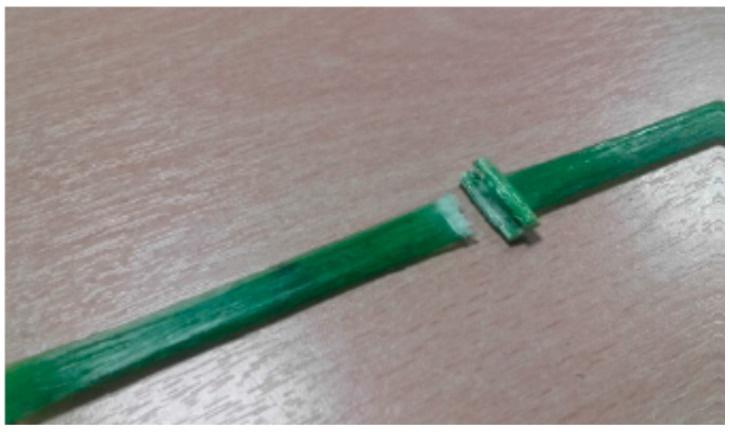
Partial fiber-failure at the GFRP grids joint.

SC specimens exhibited an average tensile strength of 986.2 MPa (SD = 85.9 MPa) and a Young’s modulus of 74,224 MPa (SD = 2904 MPa). SR-type presents a tensile strength before treatment of 1179.1 MPa (SD = 78.22 MPa) and a Young’s modulus of 70,189 MPa (SD = 1422 MPa). Two different failure modes for untreated GFRP specimens were observed: The first was a catastrophic collapse (tensile failure of the specimen approx. in the central part) (Figure 9) and the second was a partial fiber-failure at the GFRP grid joint (Figure 10).

The joint between the fibers in the two directions represents a defect as the fibers have to deviate from a straight line and rupture may easily occur in this point. Additionally, the twisted configuration in the warp direction (SC specimens) may cause a misalignment between the direction of the tensile load and the fiber orientation, reducing the tensile capacity of the specimens. Test results demonstrated that tensile strength in the warp direction (SC specimens) is approx. 16.4% smaller compared to the one in the weft direction (SR specimens). Test scattering (i.e., standard deviation, SD) is also larger for SC specimens. 

### 4.2. Treated Specimens

The attack by OH-ions is a possible degrading agent for glass fiber. In the 66 × 66 mm^2^ GFRP a thermosetting epoxy vinyl ester resin matrix was used to act as a barrier, defending the glass fibers from damaging agents. The two different types of GFRP samples (SR and SC) were subjected to ageing treatment in order to evaluate potential decrements in the mechanical characteristics after different exposure time. Two different environments were adopted to simulate exposure of specimens to field conditions: NaCl solution and deionized water solution. Specimens were removed from the ageing solutions and left to dry at room temperature for one day before testing.

#### 4.2.1. NaCl Solution

The first ageing treatment was carried out storing the specimens in NaCl solution in order to simulate aggressive environment. The quantity of NaCl added was 35 g for 1 L of deionized water. The chemical attraction between the Na^+^ and Cl^−^ ions is strong and only highly polar solvents (such as water) can dissolve completely NaCl. When dissolved in water, NaCl framework disintegrates as the Na^+^ and Cl^−^ ions become surrounded by the molecules of water. A total of 48 specimens have been tested in tension after having being treated in NaCl solution for different time lengths, up to a maximum of 15 months. 

#### 4.2.2. Deionized Water Solution

The second ageing treatment was carried out storing the specimens in deionized distilled water in order to simulate an environment characterized by high humidity. Prolonged saturation with water can affect the composite formed in the hardening process turning some into acids. These acids can break down the bond between the glass reinforcing and the resin. Deionized water is obtained by removing, with the using a series of different filters, almost all of its mineral ions (such as calcium, sodium, iron, copper and chloride). Thirty-seven GFRP samples have been tested in tension after the ageing treatment in order to evaluate the reduction of mechanical characteristics, for different treatment periods up to 15 months.

#### 4.2.3. Test Results

**Figure 11:**
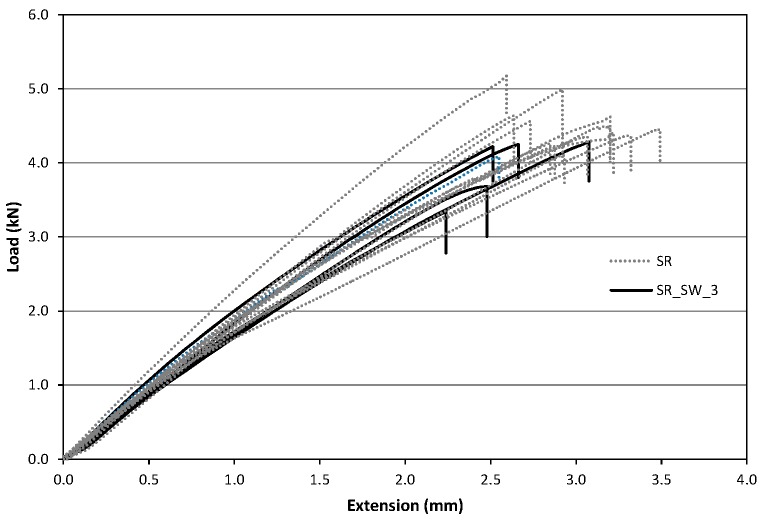
Comparison between SR and SR_SW_3.

**Figure 12:**
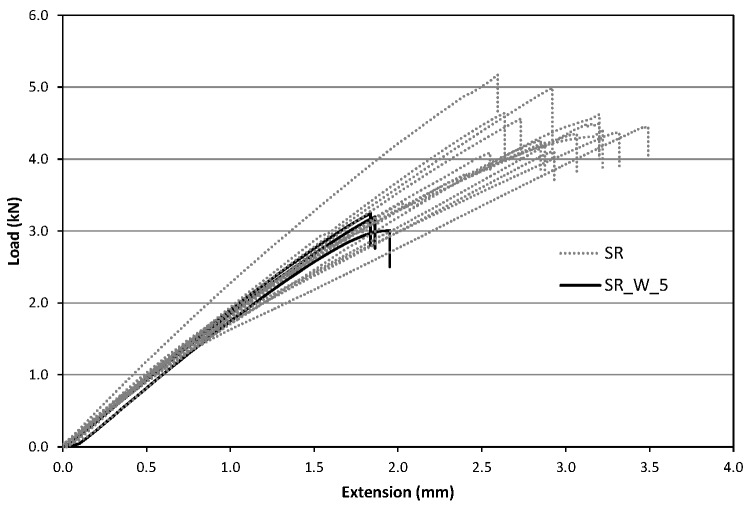
Comparison between SR and SR_W_5.

After the treatment’s time, samples were tested in tension to evaluate the possible effects on the tensile capacity and on Young’s modulus. Tests have been carried out using the same test set up introduced for the untreated specimens (Section 4.1). Similar to the previous tests, the GFRP specimens subjected to ageing treatment exhibited an approximately linear trend up to the failure similar to the untreated samples (Figure 11 and Figure 12). 

Table 5 and Table 6 show the results in terms of tensile strength and Young’s modulus for ageing treatments in NaCl and water solution, respectively: Letter designations SW and W indicate treatment in NaCl solution and in deionized water, while the number after this index indicates the treatment time in months.

From the test results it can be noted that the Young’s modulus seems not to be affected by the ageing treatments. In fact, the obtained values are similar to the those recorded for the untreated specimens. This may be because the modulus of a glassy polymer is relatively insensitive to changes in molecolar weight [34].

Specimens characterized by the circular cross-section (SC) exhibited the maximum decrease in average tensile strength after 3 and 5 months respectively for NaCl and deionized water treatments. Strength reduction was 21.2% for specimens immersed in NaCl solution and 19.4% for those in deionized water. For those characterized by rectangular cross-section (SR) the maximum decrease for the average tensile strength were 22.8% (after 7 months) and 30.2% (after 5 months) for NaCl and deionized water treatments, respectively. Figure 11 shows the average values of the tensile strength for the specimens of the SR-seires subjected to NaCl and deionized water treatments. Figure 12 shows the results for SC-samples. 

**Figure 13:**
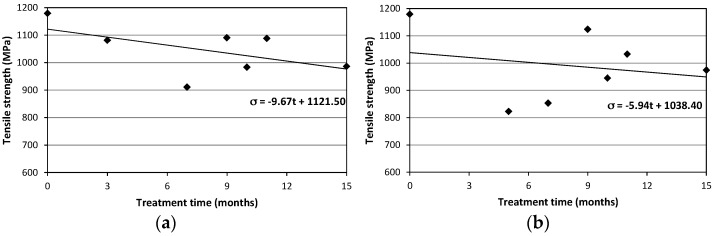
Tensile strength vs. treatment time for SR-series (**a**) NaCl solution; (**b**) deionized water.

**Figure 14:**
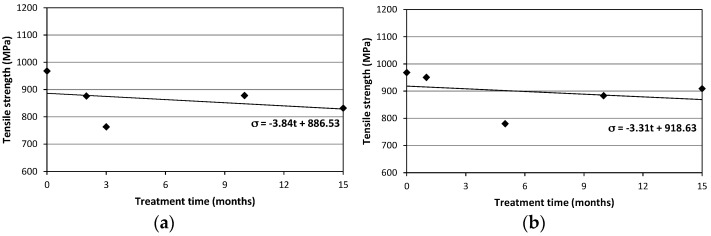
Tensile strength vs. treatment time for SC-series: (**a**) NaCl solution; (**b**) deionized water.

The Coefficients of Variation (CoV), reported in brackets in Table 5 and Table 6, are greater for SC specimens. This is again due to the non-perfect straight profile of these specimens, with the 2 cords twisted together and the seam at the interception between the cords in the two directions acting essentially as a defect able to produce a premature failure when subjected to a tensile load. Figure 13 and Figure 14 show the tensile strength vs. treatment time plots: since the number of specimens tested was limited, results should be confirmed by a larger experimental program. However, the emerging line seems quite clear and a reduction of the tensile strength has been always recorded for treated specimens. Figure 13 and Figure 14 also report a linear trend line: for both specimen and treatment types the slopes of the trend lines are negative (varying between −3.31 and −9.67). Based on the large scattering of the results, these results should be considered with high caution. It is also important to note that the strength retention is high: Over a long treatment period of 15 months the tensile strength reduction is only 6.1% and 17.4% for SC and SR specimens. This value can be considered low for a glass fiber-based composite. 

**Figure 15:**
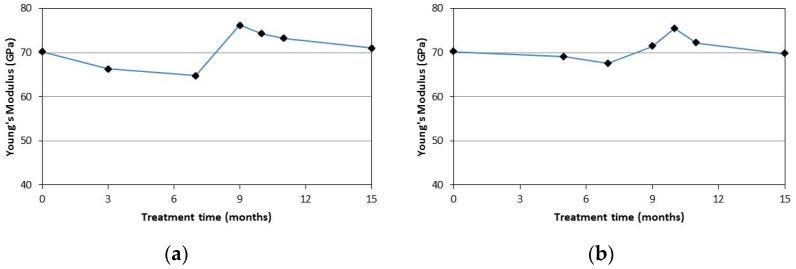
Young’s modulus vs. treatment time for SR-series (**a**) NaCl solution; (**b**) deionized water.

**Figure 16:**
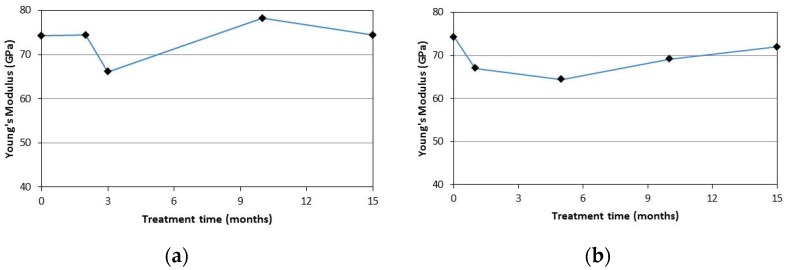
Young’s modulus vs. treatment time for SC-series: (**a**) NaCl solution; (**b**) deionized water.

Figure 15 shows the average values of the Young’s modulus for the specimens of the SR-series for both the ageing treatments after different months. Figure 16 shows the results for SC-series samples. It can be noted that a clear trend cannot be identified for the Young’s modulus. The Young’s modulus seems not to be affected by both the treatments (NaCl solution and deionized water solution) and its values remain constant with increasing treatment periods.

**Figure 17:**
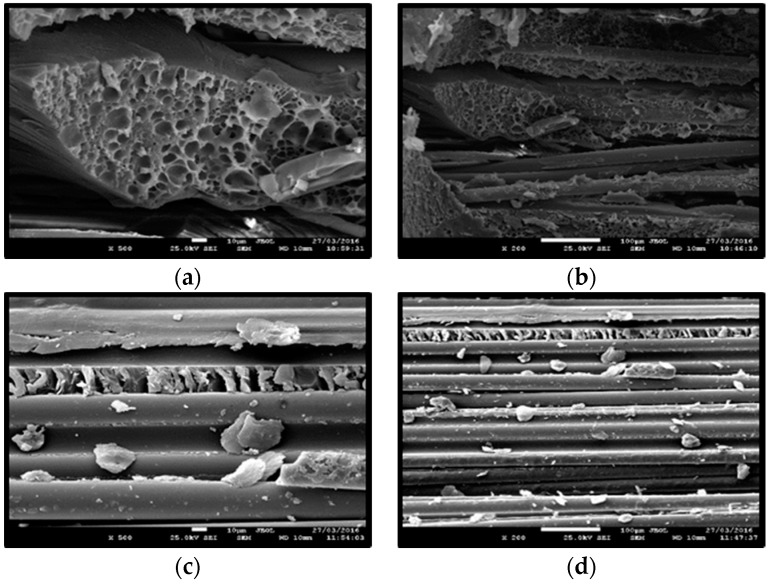
SEM micrograph of GFRP specimen aged in deionized water for 11 months: (**a**,**b**) section perpendicular to the fiber; (**c**,**d**) section parallel to the fiberglass filament.

A FEI Quanta 200 Scanning Electron Microscopy (SEM, FEI, Hillsboro, OR, USA) was used to evaluate the morphology of the fractured surfaces of the GFRP samples. Before the scanning a thin layer (15 nm thickness) of gold-palladium as conducting material has been applied on the GFRP specimen’s surface, because the untreated sample is not a conducting material and specimens could not be analyzed without the applied layer. Figure 17a,b shows SEM micrographs in a SR_W_11 specimen after failure. It can be noted the cross section of a single glass filament. Scanning electron micrographs also display micro-cracking on the fibers due to ageing in deionized water, degradation of the epoxy matrix and evidence of fibers pull out. Figure 17c,d show the glass filament before the test: It can be noted that the resin does not protect the lateral surface of the glass filaments and this is the main cause of the degradation of the mechanical characteristics of the fibers.

## 5. Conclusions

To meet the requirements of conservation bodies, apply sustainable restoration methods and to increase the durability of the bond between composite materials and masonry substrate, new retrofitting techniques have been recently proposed. In this context the use of composite materials combined with inorganic matrices is an interesting solution. This study first addressed the possible fields of application and then analyzed the durability behavior of GFRP grids exposed to various environmental conditions. 

Non-inorganic matrices made of inorganic mortars can be effectively used to bond GFRP grids to masonry substrate producing an interesting enhancement of the mechanical behavior of masonry structural members. This paper has summarized previous research conducted by the authors on the use of GFRP grids coupled with lime- or low cement-based mortars. Results of tests on wall panels reinforced using GFRP grids embedded into inorganic mortars have demonstrated that is possible to increase the lateral capacity of these structural elements, particularly vulnerable to seismic loads. 

Reinforcement at extrados of tiled vaults is another possible field of application of TRMs. The behavior of the vaults, especially the failure mode, was highly influenced by the application of the GFRP grid reinforcement. High increases in the load capacity have recorded after reinforcement. 

Masonry ring beams can be effectively constructed using recycled or new stones and bricks reinforced inside the mortar joints with GFRP grids. Test results of previous investigations carried out by the authors have been concisely reported in this paper to illustrate this.

A series of experimental tests were performed in order to obtain insight into GFRP degradation mechanisms upon prolonged exposure to ageing treatments. A total of 107 GFRP specimens have been tested in tension. Test specimens have been treated with a deionized water and NaCl solution for a duration of 15 months. Specimens immersed in deionized water show a limited decrease (6.1% and 17.4% for SC and SR specimens, respectively) in tensile strength over the 15-month period of immersion. It is seen that immersion in deionized water causes a limited decrease in the tensile strength. The treatment in deionized water did not cause any significant reduction of the elastic modulus (Young’s Modulus).

The weft and warp configuration of the GFRP grid can be problematic: in the warp direction (SC specimens) the non-straight orientation of the 2 twisted cords can produce premature failures. Also the joint between the cords in the weft and warp direction is a weak link able to cause premature tensile failures.

Tensile tests showed that GFRP specimens had a maximum reduction of tensile properties of approx. 22.8% after immersion in a NaCl solution for seven months. Test results are in line with researchers reports of degradation of the GFRP rebar or sheets varying from 10% to 47% depending upon the parameters selected for durability tests, i.e., alkalinity, moisture, temperature, stress and duration of the tests. The application of these materials for masonry retrofitting is not highly affected by this behavior in consideration on the low stress level typical of masonry structures.

## Figures and Tables

**Table 1:** Tests on wall panels.

Panel Designation	Wall Thickness (cm)	Failure Load (kN)	Shear Strength (MPa)	Shear Modulus (MPa)	Strength Increment *
CD-02-U-OR	48	31.2	0.018	29	-
CD-06-U-OR	60	44.1	0.021	35	-
CD-09-S-OR	28	19.6	0.020	150	-
CD-07-U-IP	57	333.4	0.162	2787	9.0
CD-08-U-IR	56.5	422.3	0.209	2458	11.6
CD-10-U-IR	70	543.6	0.214	-	10.2
CD-11-S-IP	38	112.1	0.086	795	4.3
CD-11-A-OR	62	53.0	0.023	83	-
CD-12-P-IP	72	215.8	0.083	668	4.1
CD-13-P-IP	64	269.2	0.117	732	5.1
CD-14-P-IP	64	204.1	0.089	895	3.8
SC-16-U-OR	48	36.1	0.032	-	-
SC-17-U-OR	60	36.7	0.023	-	-
SC-18-S-OR	28	40.6	0.062	-	-
SC-19-U-IP	67	131.6	0.116	-	5.0
SC-20-U-IP	56.5	203.8	0.236	-	7.4
SC-21-U-IR	56.5	155.3	0.173	-	5.4

* ratio between shear strength of reinforced and unreinforced panels.

**Table 2:** Test results on masonry arches.

Arch Designation	Mortar Type	Failure LOAD (kN)	Load Point Deflection (mm)	Failure Mode
DR.UT.01	1	0.15	-	Mechanism
DR.UT.02	2	1.57	2.46	Mechanism
DR.IT.01	1	0.82	4.88	Ring separation + snap-through buckling
DR.IT.02	2	3.52	26.53	Ring separation + snap-through buckling
DR.OT.01	2	8.32	27.13	Ring separation + snap-through buckling
DR.OT.02	2	10.54	3.21	Ring separation + snap-through buckling
DR.OT.03	2	8.50	13.93	Ring separation + snap-through buckling
DR.OT.04	2	7.49	8.64	Ring separation + snap-through buckling
TR.UT.01	1	1.07	1.34	Mechanism
TR.UT.02	2	6.83	4.93	Mechanism
TR.UT.03	2	4.10	7.69	Mechanism
TR.IT.01	1	2.71	2.23	Ring separation + snap-through buckling
TR.IT.02	2	8.46	17.60	Ring separation + snap-through buckling
TR.IT.03	2	12.99	24.79	Ring separation + snap-through buckling
TR.OT.01	2	16.66	14.04	Ring separation + snap-through buckling
TR.OT.02	2	17.83	13.06	Ring separation + snap-through buckling
TR.OT.03	2	13.16	9.90	Ring separation + snap-through buckling

**Table 3:** Test results on ring beams.

Beam Designation	Dry Fiber cross Sectional Area (mm^2^)	Bending Moment *M*_W_ (kNm)	Failure Bending Load (kN)	Bending Moment *M*_Load_ (kNm)	Total Mending Moment (kNm)
P5-G33-V	171.1	10.7	56.8	39.75	50.45
P6-G33-H	171.1	10.7	56.6	39.62	50.32
P7-G66-V	342.2	10.7	62.9 ^+^	44.03	54.73
P8-G66-H	342.2	10.7	43.1 ^+^	30.14	40.84
L9-G33-V	213.9	3.86	51.2	35.87	39.73
L10-G33-H	213.9	3.86	38.3 ^+^	26.80 ^+^	30.66

^+^ beam failure not reached.

**Table 4:** Mechanical characteristics of the untreated GFRP specimens (SD = standard deviation).

Index (-)	No. of Specimens (-)	Max Load (N)	Tensile Strength (MPa)	Young’s Modulus (MPa)	Ultimate Strain (%)
SC	7	3679	986.2 (85.93) *	74,224	1.30
SR	15	4480	1179.1 (78.22) *	70,189	1.68

* standard deviation.

**Table 5:** Test results for GFRP specimens subjected to ageing treatment (NaCl solution).

Index	No. of Specimens	Exposure Time (Months)	Maximum Load (N)	Tensile Strength (MPa)	Young’s Modulus (MPa)	Strength Decrease (%)	Young’s Modulus Decrease (%)
SC	7	0	3679	968.2 (9.6%)	74,224	-	-
SC_SW_2	3	2	3329	876.2 (2.4%)	75,775	9.5	−2.09
SC_SW_3	3	3	2900	763.3 (3.2%)	66,060	21.2	11.0
SC_SW_10	3	10	3337	878.1 (14.3%)	78,180	9.3	−5.33
SC_SW_15	3	15	3160	831.6 (15.2%)	76,287	14.1	−2.78
SR	15	0	4480	1179 (6.6%)	70,189	-	-
SR_SW_3	5	3	4108	1081 (6.9%)	66,329	8.3	5.55
SR_SW_7	7	7	3459	910.4 (11.5%)	64,793	22.8	7.68
SR_SW_9	7	9	4142	1090.2 (6.4%)	76,204	8.1	−8.57
SR_SW_10	7	10	3682	983.1 (7.3%)	74,316	16.6	−5.88
SR_SW_11	7	11	4132	1088.0 (4.3%)	73,284	7.7	−4.41
SR_SW_15	3	15	3747	986.3 (3.7%)	71,066	16.3	−1.25

CoV in ( ).

**Table 6:** Test results for GFRP specimens subjected to ageing treatment (distilled water).

Index	No. of Specimens	Exposure Time (Months)	Maximum Load (N)	Tensile Strength (MPa)	Young’s Modulus (MPa)	Strength Decrease (%)	Young’s Modulus Decrease (%)
SC	7	0	3679	968.2 (9.6%)	74,224	-	-
SC_W_1	3	1	3610	950.3 (4.7%)	67,025	1.8	9.7
SC_W_5	3	5	2964	780.1 (6.1%)	64,427	19.4	13.2
SC_W_10	3	10	3355	882.9 (15.7%)	69,103	8.8	6.9
SC_W_15	3	15	3454	909.1 (14.4%)	71,923	6.1	3.1
SR	15	0	4480	1179 (6.6%)	70,189	-	-
SR_W_5	3	5	3125	822.5 (5.5%)	69,031	30.2	1.65
SR_W_7	10	7	3241	853.1 (9.3%)	67,501	27.7	3.83
SR_W_9	3	9	4270	1123.8 (4.6%)	71,389	4.7	−1.71
SR_W_10	3	10	3590	944.9 (4.2%)	75,453	19.9	−7.5
SR_W_11	3	11	3924	1032.8 (6.1%)	72,224	12.4	−2.9
SR_W_15	3	15	3701	974.1 (3.7%)	69,775	17.3	0.59

CoV in ( ).
